# Behavioral stress alters corticolimbic microglia in a sex- and brain region-specific manner

**DOI:** 10.1371/journal.pone.0187631

**Published:** 2017-12-01

**Authors:** Justin L. Bollinger, Kaitlyn E. Collins, Rushi Patel, Cara L. Wellman

**Affiliations:** 1 Department of Psychological and Brain Sciences, Indiana University, Bloomington, IN, United States of America; 2 Program in Neuroscience, Indiana University, Bloomington, IN, United States of America; 3 Center for the Integrative Study of Animal Behavior, Indiana University, Bloomington, IN, United States of America; Uniformed Services University, UNITED STATES

## Abstract

Women are more susceptible to numerous stress-linked psychological disorders (e.g., depression) characterized by dysfunction of corticolimbic brain regions critical for emotion regulation and cognitive function. Although sparsely investigated, a number of studies indicate sex differences in stress effects on neuronal structure, function, and behaviors associated with these regions. We recently demonstrated a basal sex difference in- and differential effects of stress on- microglial activation in medial prefrontal cortex (mPFC). The resident immune cells of the brain, microglia are implicated in synaptic and dendritic plasticity, and cognitive-behavioral function. Here, we examined the effects of acute (3h/day, 1 day) and chronic (3h/day, 10 days) restraint stress on microglial density and morphology, as well as immune factor expression in orbitofrontal cortex (OFC), basolateral amygdala (BLA), and dorsal hippocampus (DHC) in male and female rats. Microglia were visualized, classified based on their morphology, and stereologically counted. Microglia-associated transcripts (CD40, iNOS, Arg1, CX3CL1, CX3CR1, CD200, and CD200R) were assessed in brain punches from each region. Expression of genes linked with cellular stress, neuroimmune state, and neuron-microglia communication varied between unstressed male and female rats in a region-specific manner. In OFC, chronic stress upregulated a wider variety of immune factors in females than in males. Acute stress increased microglia-associated transcripts in BLA in males, whereas chronic stress altered immune factor expression in BLA more broadly in females. In DHC, chronic stress increased immune factor expression in males but not females. Moreover, acute and chronic stress differentially affected microglial morphological activation state in male and female rats across all brain regions investigated. In males, chronic stress altered microglial activation in a pattern consistent with microglial involvement in stress-induced dendritic remodeling across OFC, BLA, and DHC. Together, these data suggest the potential for microglia-mediated sex differences in stress effects on neural structure, function, and behavior.

## 1. Introduction

Women are more vulnerable to various stress-linked psychopathologies, including depression, most anxiety disorders, and post-traumatic stress disorder [1–5]. Structural and functional alterations in medial prefrontal cortex (mPFC), orbitofrontal cortex (OFC), basolateral amygdala (BLA), and dorsal hippocampus (DHC) have been implicated in these disorders [6, 7], and stress-induced changes in these regions are associated with disease-relevant behaviors in rodent models, including working memory dysfunction [8], anhedonia [9], and anxiety-like behavior [10].

Stress alters many of these structures in a sex- and region-specific manner. For instance, chronic restraint stress induces apical dendritic retraction in pyramidal neurons in mPFC and DHC in male rats, but little to no change or even dendritic growth in females [11–14]. These differences in neuronal remodeling correspond to differences in behavior: males typically show chronic stress-induced deficits in memory-associated tasks whereas females do not [8, 15–17].

As the resident immune cells of the central nervous system, microglia monitor the brain microenvironment, perisynaptic contacts, and dendritic spines for pathogens, debris, or cellular damage [18, 19]. When activated, microglia transition through several neuromodulatory states [20]. Activated microglia can reorient their processes toward neuronal and astroglial signals (e.g. glutamate, extracellular purines) [21], and regulate neuronal function through release of neuroactive factors (e.g., inflammatory cytokines and reactive oxygen species), direct pruning of dendritic spines [22], and reorganization of dendritic architecture [23].

In male rats, chronic stress induces morphological activation of microglia in mPFC, medial amygdala, and DHC, among other regions [24, 25], and primes multiple structures toward a pro-inflammatory state [26, 27]. These alterations in microglial morphology and immune factor expression vary in magnitude, are region- and stressor-specific, and correlate with behavioral deficits [24, 25, 28, 29]. We recently demonstrated a dramatic sex difference in measures of microglial activation in mPFC in unstressed rats, as well as sex-dependent effects of stress on microglial activation in mPFC [30]. However, mPFC is but one critical node in a network of regions involved in regulating emotion and cognition, and stress alters brain architecture and function in a stressor- and region-specific manner. For instance, in male rats, chronic stress induces dendritic loss in mPFC and DHC [13, 31], but dendritic growth in OFC and BLA [32]. Therefore, to begin to more thoroughly characterize potential sex- and brain region-dependent stress effects on corticolimbic microglia, we assessed microglial morphology and immune factor expression in OFC, BLA, and DHC in males and females following acute and chronic restraint stress. Identifying different patterns of stress-induced changes across these corticolimbic brain regions is critical to a circuit-level understanding of how stress influences emotional and cognitive behaviors.

We analyzed a number of immune factors that may be differentially expressed between males and females, and are implicated in neuron-microglia communication and neural plasticity. This included the antigen presentation-associated molecule cluster of differentiation (CD) 40 (a marker of microglial or neuroimmune activation) [33]. Expression of CD40 is differentially affected by sex and stress in mPFC [30]. Moreover, the ligand for CD40 (CD40L) is located on the X chromosome and is thus subject to X-inactivation in female but not male rats. This may allow for the differential expression of CD40L-CD40 signaling across microglia in females, and may contribute to sex differences in basal and stress-induced CD40 expression and microglial activation [34]. Inducible nitric oxide synthase (iNOS) and arginase-1 (Arg1, indicators of oxidative stress/potential microglial pro-inflammatory function and anti-inflammatory function, respectively) were also examined [35, 36]. It should be noted that iNOS and Arg1 compete for the same amino acid (L-arginine), and thus Arg1 can regulate the function of iNOS by acting on this substrate, a direct precursor to the synthesis of nitric oxide. Given this interaction, upregulated iNOS concurrent with heightened Arg1 may reflect neither an anti- or pro-inflammatory profile, but rather broad neuroimmune state or a mixed microglial phenotype [37]. The chemokine, fractalkine (CX3CL1) and its cognate receptor (CX3CR1) were analyzed, as neuronally expressed CX3CL1 may modulate glutamatergic tone, inhibit microglial cell activation, and regulate the effects of chronic stress on neuronal plasticity and depressive-like behaviors through microglial CX3CR1-mediated actions [38, 39]. Neuronal and astroglial CD200 and microglial CD200R were also assessed, as direct CD200-CD200R interaction can regulate microglial surveillance, inhibit microglial activation, and reduce pro-inflammatory cytokine expression [40, 41]. These chemokine/costimulatory pathways were examined in lieu of canonical cytokines given their membrane bound location (neurons, CX3CL1 and CD200; microglia, CX3CR1 and CD200R), potential soluble form (CX3CL1 and CD200), and involvement in microglial chemotaxis and process maintenance [42], both functions implicated in morphological remodeling and direct neuron-microglia interaction.

## 2. Experimental procedures

### 2.1 Animal manipulations and restraint stress

Brain sections (see Section 2.2) and punches (see Section 2.3) were obtained from the same male and female Sprague-Dawley rats used for previous analyses of mPFC; see [30]. Animals were group-housed by sex and stress condition (3 rats/cage). Rats underwent either acute (1 day; Male: n = 23, Female: n = 20) or chronic (10 consecutive days; Male: n = 24, Female: n = 21) restraint stress, or were left unstressed (Male: n = 23, Female: n = 20). Restraint stress was performed by placing rats in a clear plastic semi-cylindrical rodent restrainer in their home cages under bright lights for 3 hours/day. Restrainers were identical in form with two bore sizes (Male: 16 cm × 6.5 cm × 5 cm, Female: 15 cm × 6 cm × 4.5 cm) and were adjustable in length, allowing for comparable restraint across differing animal weights. The time of day during which restraint occurred varied in an unpredictable manner over the light phase of the light-dark cycle (i.e. time of stress; day 1: 1000 h; day 2: 1600 h; day 3: 0900 h; etc.). This procedure decreases habituation to the stressor and produces significant increases in plasma corticosterone [13, 31], adrenal hypertrophy [11], sex differences in dendritic remodeling [11, 14], and sex specific patterns of microglial activation [30].

Estrous phase was determined as previously reported [30]. In brief, vaginal cytology was examined post-restraint, just prior to euthanasia between 1100 and 1630 h. Given limited representation across estrous phases and stress conditions (diestrus: n = 54; proestrus: n = 1 unstressed, 2 acute stress, 3 chronic stress; estrus: n = 1 chronic stress), we did not analyze our data relative to estrous cycle. On the final day of restraint, animals were euthanized 1.68 ± 0.11 h post-stress and brains were processed for either immunohistochemical visualization of microglia (section 2.2) or quantitative real-time PCR (qPCR) for immune factor expression (section 2.3). All procedures were performed during the light phase, carried out in accordance with the *NIH Guide for the Care and Use of Laboratory Animals*, and approved by the Bloomington Institutional Animal Care and Use Committee.

Chronic stress can reduce weight gain and induce adrenal hypertrophy in male and female rats [43]. Therefore, to verify stressfulness of the manipulation, adrenals were dissected and weighed. Adrenal-weight-to-body-weight ratios and rate of weight gain [(body weight at end of experiment—body weight at start) / number of experimental days] were compared across groups.

### 2.2 Effects of stress on microglial cell morphology in OFC, BLA, and DHC

Microglia were immunohistochemically visualized as previously described [30], and cellular density and morphology were analyzed in OFC, BLA, and stratum radiatum and stratum oriens of the CA3 subregion of dorsal hippocampus (DHC).

#### 2.2.1 Iba-1 immunohistochemistry

Animals (unstressed: male n = 13, female n = 10; acute stress: male n = 13, female n = 10; chronic stress: male n = 14, female n = 11) were overdosed with urethane and transcardially perfused with 0.1 M phosphate-buffered saline (PBS) followed by 4% paraformaldehyde in PBS. Brains were removed, postfixed overnight, cryoprotected, and sectioned. Free-floating coronal sections (44μm) were stained with an antibody specific to ionized calcium-binding adaptor protein-1 (iba-1, 1:1000; Wako Chemicals Inc., Richmond, VA). Iba-1 is constitutively expressed in microglia, involved in cytoskeletal reorganization, and up-regulated in response to microglial cell activation [44]. Immunopositive cells were visualized with a Ni-intensified DAB reaction, and sections were counterstained with neutral red to facilitate identification of regions of interest.

#### 2.2.2 Stereology

**Fig 1:**
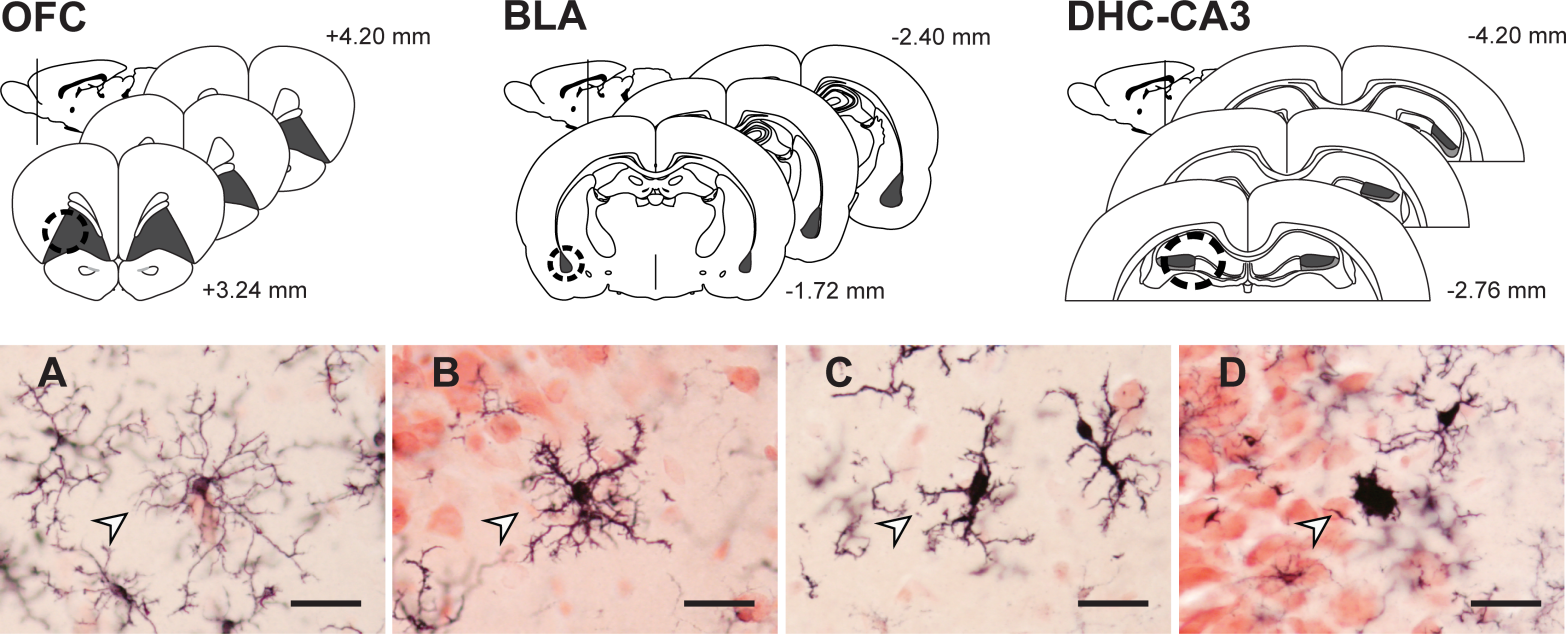
Corticolimbic sampling and microglial cell morphology. Stereological estimates (shaded in grey) and micropunch samples (dashed circle, indicated in one hemisphere for simplicity) were derived from both hemispheres of OFC, BLA, and DHC (CA3 stratum radiatum, dark grey; CA3 stratum oriens, light grey) using standard gross morphology (micropunch) and cytoarchitecture (stereology) [45]. Microglia were classified as A) surveillant (numerous thin processes, radial branching); B) primed (thickened processes, increased polarity, proliferation, some reduced secondary branching); C) reactive (thickened stout processes with highly reduced branching); or D) amoeboid (few-to-no processes, enlarged cell body). Scale bars = 10 μm. Arrowheads indicate exemplars of each type.

We analyzed microglial cell morphology and density in the left and right hemispheres of OFC (3–4 sections per animal), BLA (4–6 sections per animal), and the CA3 subregion of DHC (5–6 sections per animal, Fig 1). Sampling occurred throughout the anterior-posterior axis of each region [45]. Cells were counted at a final magnification of 1800× using the optical fractionator method and StereoInvestigator (MicroBrightField Inc., Williston, VT). Microglia were classified as surveillant (numerous thin processes, radial branching), primed (thickened processes, increased polarity, reduced secondary branching), reactive (thickened stout processes with highly reduced branching), or amoeboid (rounded soma with little to no branching) based on standard morphological criteria (Fig 1) [46, 47]. Very few reactive (OFC: < 3% of cells; BLA: < 1%; CA3 stratum: < 3%; CA3 oriens: < 4%) or amoeboid (OFC: < 1% of cells; BLA: < 1%; CA3 stratum: < 2%; CA3 oriens: < 2%) microglia were present. Therefore, analyses focus on surveillant and primed microglia, with subtype proportions expressed as the ratio of primed cell density to surveillant cell density [47]. In OFC, approximately 739 cells per animal were counted [grid size (GS): 400 × 500; counting frame (CF): 150^2^ μm; mean CE: 0.05]; 329 cells were counted in BLA (GS: 185 ^2^; CF: 150^2^ μm; mean CE: 0.06); in stratum radiatum and stratum oriens of CA3, 182 cells (GS: 350^2^; CF: 150^2^; mean CE: 0.10) and 96 cells (GS: 350^2^; CF: 150^2^ μm; mean CE: 0.14) were counted, respectively. Guard zones were set with a centered-probe thickness of 10 μm for each region. Counts were performed blind to sex and experimental condition. Estimated densities and relative proportions were calculated for each cell type. Statistical tests consisted of 2-way ANOVAs. Significant effects were followed by post-hoc comparisons (Fisher’s protected LSD; IBM SPSS Statistics 24, IBM Corp., Armonk, NY).

Given that repeated HPA axis activation increases adrenal weights, associations between adrenal-weight-to-body-weight ratios and microglial- density and morphology were examined in unstressed rats and rats exposed to chronic stress using Pearson correlation coefficients. Rats exposed to acute stress were excluded from these analyses, as acute stress can affect microglial morphology but not adrenal weight. In addition, Pearson correlations were used to examine the relationships among microglial morphological activation in OFC, BLA, DHC, and mPFC. For this analysis, data for mPFC were obtained from [30]. These data were included because mPFC is an important node in the corticolimbic circuitry underlying cognition and emotion. Correlations were analyzed separately by sex and group. Outliers more than ±2.5 standard deviations from the mean were removed from all analyses.

### 2.3 Effects of stress on immune factor expression in OFC, BLA, and DHC

Expression of CD40, iNOS, Arg1, CX3CL1, CX3CR1, CD200, and CD200R in OFC, BLA, and DHC was analyzed using qPCR following the final session of restraint.

#### 2.3.1 Tissue collection, RNA isolation, and cDNA synthesis

Animals (Male: n = 10 per group, Female: n = 10 per group) were overdosed, transcardially perfused with ice-cold PBS to remove peripheral macrophages and immune molecules, and brains were rapidly extracted and snap-frozen as previously described [30]. Slices through the rostral-caudal extent of each region of interest (1–2 mm) were taken at -20°C. OFC, BLA, and DHC were identified using standard gross neuroanatomical landmarks [45] and micropunch samples (1 mm dia., approximately 3–8 mg tissue/animal) from both hemispheres of each region were collected. Total RNA was isolated using a previously described Trizol extraction technique; see [30]. RNA integrity and concentration were analyzed. Samples with an RNA Integrity Number (RIN) > 7.5 (mean RIN: 8.75) were reverse transcribed into first strand cDNA (20 μl) and stored at -20°C; see [30].

#### 2.3.2 Quantitative PCR

Primers to measure expression of each gene of interest, as well as the reference gene glyceraldehyde-6-phosphate dehydrogenase (GAPDH), were designed using the Roche Universal Probe Library (lifescience.roche.com/en_us/brands/universal-probe-library, Table 1) alongside previously described resources [30]. Primers were obtained from Eurofins Genomics (Eurofins MWG Operon LLC, Huntsville, AL). Formation of PCR product was measured in real time using the Roche LightCycler 480 System (Roche Diagnostics, Indianapolis, IN). For each sample, triplicate reactions were performed in 384-well plates as previously described [30], with the concentration of cDNA adjusted to 13.6 ng per reaction. For each qPCR assay, gene expression was undetectable in -template and -reverse transcriptase reactions. To evaluate the relative abundance of mRNA, mean C_T_ values were computed across reaction triplicates for each sample, with replicate C_T_ standard deviations above 1 removed from analysis. Relative gene expression was then quantified using the 2^-ΔΔCT^ method [48] and evaluated using two-way ANOVA; significant effects were followed by post-hoc comparisons using Fisher’s protected LSD. Differences in expression between genes were not analyzed. Outliers more than ±2.5 standard deviations from the mean were removed from analyses. See S1 File for all relevant data.

**Table 1:** qPCR primer sequences.

Gene	Primer Sequence (5' - 3')	Avg. Ct ± SEM
CD40	F—GCCACTGAGACTACTGATACTG	28.2 ± 0.1
*NM_134360*.*1*	R—TGACTTGTTCCTTCCCGTAG	
iNOS	F—GGAGCAGGTTGAGGATTACTTC	32.4 ± 0.1
*NM_012611*.*3*	R—AAAAGACCGCACCGAAGAT	
Arg1	F—AAGACAGGGCTACTTTCAGGAC	24.1 ± 0.1
*NM_017134*.*3*	R—ACCTTCCCGTTTCGTTCCAA	
CX3CL1	F—GAATTCCTGGCGGGTCAGCACCTCGGCATA	21.3 ± 0.2
*NM_134455*.*1*	R—AAGCTTTTACAGGGCAGCGGTCTGGTGGT	
CX3CR1	F—AGCTGCTCAGGACCTCACCAT	23.8 ± 0.1
*NM_133534*.*1*	R—GTTGTGGAGGCCCTCATGGCTGAT	
CD200	F—TGTTCCGCTGATTGTTGGC	21.1 ± 0.1
*NM_031518*	R—ATGGACACATTACGGTTGCC	
CD200R	F—TGCCAAAATCGGGAGCTA	30.5 ± 0.2
*AF231392*	R—AGCTAGCATACGGCTGCATT	
GAPDH	F—ACCACAGTCCATGCCATCACTG	18.3 ± 0.1
*NM_017008*.*4*	R—GATGACCTTGCCCACAGCCTT	* *

## 3. Results

### 3.1 Daily weight gain and adrenal weight

**Fig 2:**
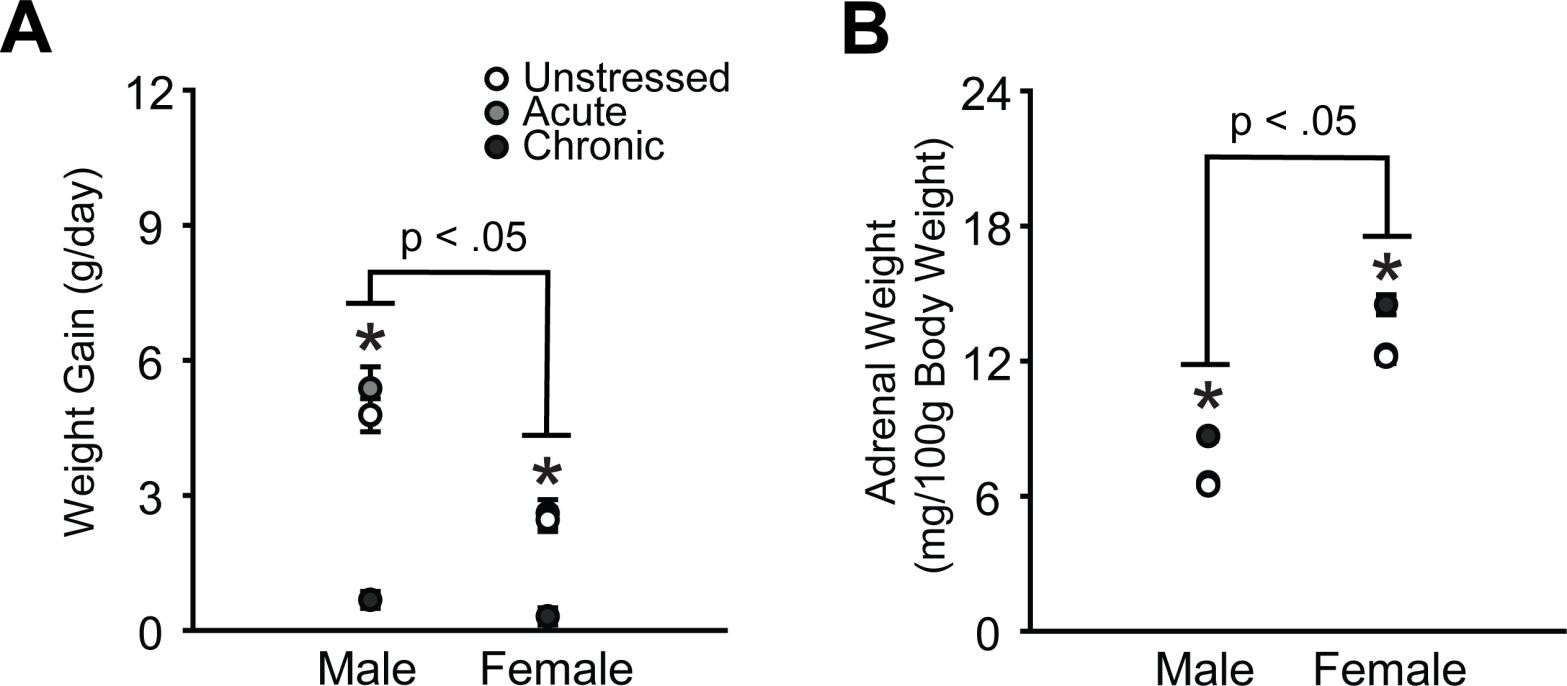
Chronic stress reduces daily weight gain and induces adrenal hypertrophy in male and female rats. A. Data are displayed as mean weight gain per day (end body weight–starting body weight/number of study days). B. Data are displayed as mean adrenal-weight-to-body-weight ratios. *p < .05 chronic stress group compared to same-sex unstressed group. Error bars indicate SEM.

Male rats gained significantly more weight per day compared to female rats (effect of sex: *F*_(1, 124)_ = 48.69, p < .001), and chronic stress reduced daily weight gain in males and females (effect of stress: *F*_(2, 124)_ = 73.57, p < .001, Fig 2A). This effect was more pronounced in male rats (sex × stress interaction: *F*_(2, 124)_ = 8.26, p < .001), likely due to the more substantial weight gain in unstressed males relative to females. Consistent with previous research [49], adrenal-weight-to-body-weight ratios were significantly higher in females, regardless of stress (effect of sex: *F*_(1, 125)_ = 713.84, p < .001, Fig 2B). Chronic stress significantly increased adrenal weights in males and females (effect of stress: F_(2, 125)_ = 43.93, p < .001). Aside from one moderate, negative association (microglial density in OFC in females: *r*_(21)_ = -0.45, p = .04), adrenal-weight-to-body-weight ratios were not significantly correlated with microglial density or morphological activation state across OFC, mPFC, BLA, CA3 radiatum, or CA3 oriens in male or female rats (S1 Table).

### 3.2 Microglial cell morphology and immune factor expression in orbitofrontal cortex

**Fig 3:**
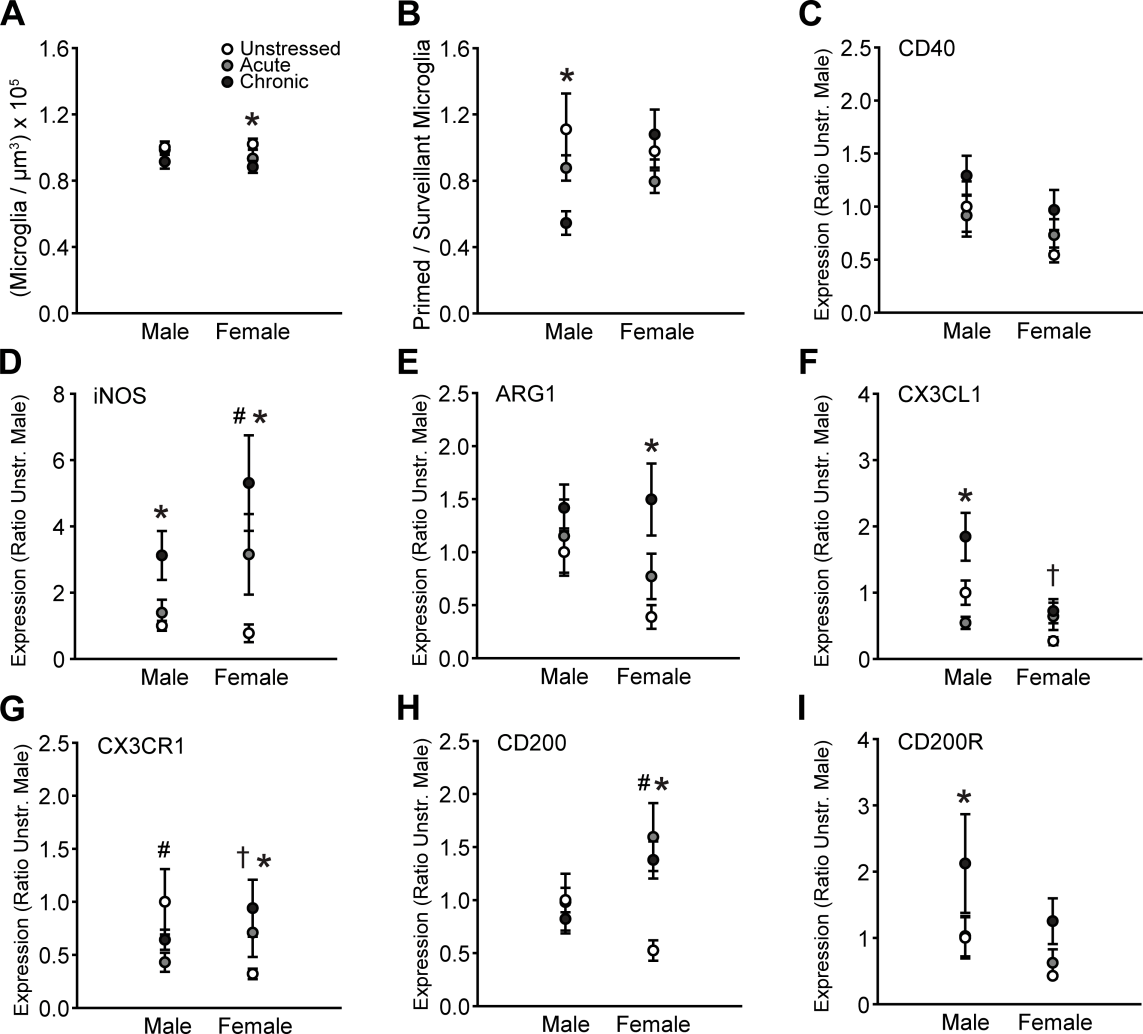
Sex and stress effects on microglial morphology and immune factor expression in orbitofrontal cortex. A. Total microglial density based on estimated volume. Chronic stress slightly decreased total microglial density in females. B. Chronic stress reduced the proportion of primed to surveillant microglia in males. C. There were no sex differences in- or stress effects on CD40 expression. D. Acute stress increased iNOS transcript in females. Chronic stress induced iNOS expression in males and females. E. Chronic stress increases Arg1 mRNA in females. F. CX3CL1 expression is lower in unstressed females compared to unstressed males. Chronic stress increased CX3CL1 mRNA in males. G. Expression of CX3CR1 is reduced in unstressed females compared to unstressed males. Acute stress increases CX3CR1 mRNA in males, whereas chronic stress increases CX3CR1 transcript in females. H. Acute and chronic stress increase CD200 expression in females. I. Chronic stress increases CD200R mRNA in males. ^†^p < .05, unstressed males compared to unstressed females. ^#^p < .05, acute stress group compared to same-sex unstressed group. *p < .05, chronic stress group compared to same-sex unstressed group. Error bars indicate SEM. For graphs C-I, data are expressed relative to unstressed males.

Total microglial density in OFC did not vary with sex (*F*_(1, 65)_ = 0.52, ns), though stress significantly altered density (*F*_(2, 65)_ = 4.53, p = .01 sex × stress interaction: *F*_(2, 65)_ = 0.47, ns). Follow-up comparisons indicate small, chronic stress-induced reductions in total microglial density in female but not male rats (p = .02, Fig 3A). Morphological analyses revealed that overall, neither sex (*F*_(1, 65)_ = 0.99, ns) nor stress (*F*_(2, 65)_ = 1.88, ns) altered the proportion of primed to surveillant microglia. However, the effect of stress varied between males and females (*F*_(2, 65)_ = 4.14, p = .02). Chronic stress markedly reduced the proportion of primed to surveillant microglia in male but not female rats (p < .01, Fig 3B).

Overall, CD40 expression differed in males and females (*F*_(1, 50)_ = 4.32, p = .04). No significant main effects of stress were detected (effect of stress: *F*_(2, 50)_ = 2.08, ns; sex × stress interaction: *F*_(2, 50)_ = 0.64, ns).

Expression of iNOS varied with both sex and stress (main effect of sex: *F*_(1, 48)_ = 3.93, p = .05; main effect of stress: *F*_(2, 48)_ = 9.86, p < .001; sex × stress interaction: *F*_(2, 48)_ = 1.47, ns). Follow up comparisons indicate that acute stress increased iNOS transcript in females (p = .04), and chronic stress increased iNOS expression in males (p = .04) and females (p < .001, Fig 3D).

Overall, Arg1 expression did not vary with sex (*F*_(1, 52)_ = 2.06, ns). However, stress significantly altered levels of Arg1 mRNA (*F*_(2, 52)_ = 4.56, p = .02; sex × stress interaction: *F*_(2, 52)_ = 0.94, ns). Chronic stress significantly increased Arg1 transcript in females but not males (p < .01, Fig 3E).

Levels of CX3CL1 mRNA varied with both sex (*F*_(1, 50)_ = 11.44, p < .01) and stress (*F*_(2, 50)_ = 6.54, p < .01), and the effect of stress on CX3CL1 expression differed between males and females (*F*_(2, 50)_ = 4.20, p = .02). Planned comparisons revealed heightened CX3CL1 transcript in unstressed males compared to unstressed females (p = .01). Chronic stress increased CX3CL1 transcript in males (p < .01) but not females.

Although CX3CR1 expression did not vary with sex (*F*_(1, 52)_ = 0.04, ns) or stress (*F*_(2, 52)_ = 0.53, ns), stress differentially altered levels of CX3CR1 mRNA in males and females (*F*_(2, 52)_ = 3.59, p = .04). Unstressed males exhibited heightened CX3CR1 expression compared to unstressed females (p = .02). Acute stress decreased CX3CR1 transcript in males (p = .05), whereas chronic stress increased CX3CR1 mRNA in females (p = .05, Fig 3G).

CD200 expression did not vary with sex (*F*_(1, 52)_ = 2.15, ns). However, stress significantly altered levels of CD200 mRNA in a sex-dependent manner (effect of stress: *F*_(2, 52)_ = 3.86, p = .03; sex × stress interaction: *F*_(2, 52)_ = 5.23, p < .01). Acute and chronic stress increased CD200 expression in females but not males (p < .01, Fig 3H). Moreover, stress altered CD200R expression (main effect of stress: *F*_(2, 50)_ = 3.33, p = .04; main effect of sex: *F*_(1, 50)_ = 3.29, ns; sex × stress interaction: *F*_(2, 50)_ = 0.16, ns). Chronic stress increased CD200R transcript in male (p = .05, Fig 3I) but not female rats.

### 3.3 Microglial cell morphology and immune factor expression in basolateral amygdala

**Fig 4:**
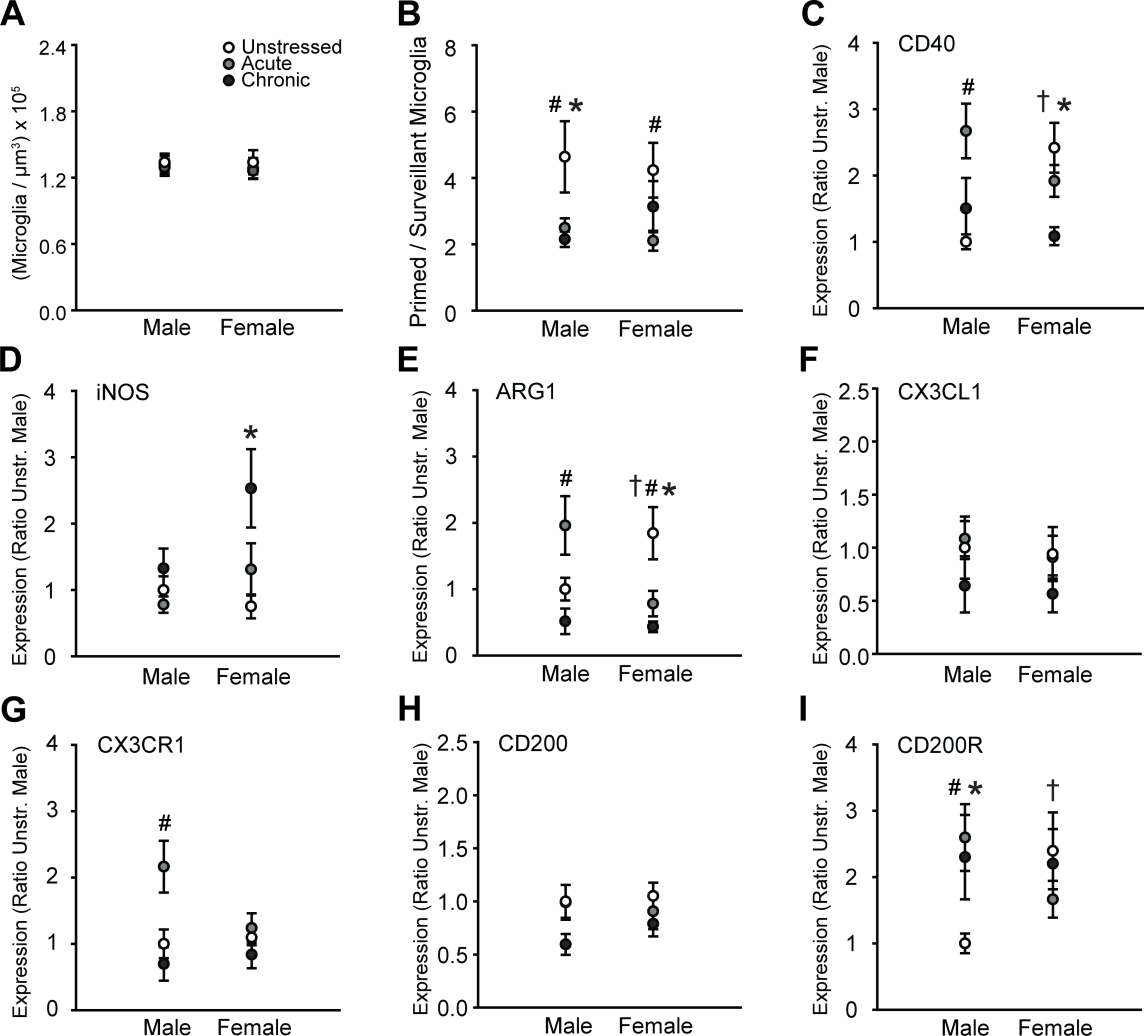
Sex and stress effects on microglial morphology and immune factor expression in basolateral amygdala. A. Total microglial density based on estimated volume. There were no effects of sex or stress on microglial density. B. Acute stress reduced the proportion of primed to surveillant microglia in males and females. Chronic stress reduced this proportion in males only. C. Expression of CD40 is significantly higher in unstressed females compared to unstressed males. Acute stress induced CD40 expression in males. Chronic stress decreased CD40 transcript in females. D. Chronic stress increased iNOS mRNA in females only. E. Unstressed females exhibited greater Arg1 expression compared to unstressed males. Acute stress induced Arg1 expression in males, whereas acute and chronic stress reduced Arg1 transcript in females. F. CX3CL1 expression did not vary with sex or stress. G. Acute stress increased CX3CR1 transcript in males only. H. There were no effects of sex or stress on CD200 mRNA. I. CD200R expression is higher in unstressed females compared to unstressed males. Acute and chronic stress increase CD200R transcript in males only. ^†^p < .05, unstressed males compared to unstressed females. ^#^p < .05, acute stress group compared to same-sex unstressed group. *p < .05, chronic stress group compared to same-sex unstressed group. Error bars indicate SEM. For graphs C-I, data are expressed relative to unstressed males.

In BLA, total density of microglia did not vary across groups (effect of sex: *F*_(1, 65)_ = 1.21, ns; effect of stress: *F*_(2, 65)_ = 1.13, ns; sex × stress interaction: *F*_(2, 65)_ = 0.16, ns; Fig 4A). However, morphological analyses revealed a significant effect of stress on the proportion of primed to surveillant microglia (main effect of stress: *F*_(2, 65)_ = 6.30, p < .01; main effect of sex: *F*_(1, 65)_ = 0.08, ns; sex × stress interaction: *F*_(2, 65)_ = 0.62, ns). Acute stress decreased the proportion of primed to surveillant microglia in males (p < .01) and females (p = .04), whereas chronic stress reduced the proportion of primed to surveillant microglia in males only (p < .01, Fig 4B).

Stress altered CD40 expression (*F*_(2, 54)_ = 4.66, p = .01), and this effect differed between males and females (effect of sex: *F*_(1, 54)_ = 0.10, ns; sex × stress interaction: *F*_(2, 54)_ = 7.16, p < .01). CD40 expression was greater in unstressed females compared to unstressed males (p < .01). Acute stress increased CD40 expression in males only (p < .001), whereas chronic stress decreased levels of CD40 mRNA in females but not males (p < .01, Fig 4C).

There was a significant main effect of stress on iNOS expression (effect of stress: *F*_(2, 53)_ = 6.40, p < .01; effect of sex: *F*_(1, 53)_ = 3.67, ns; sex × stress interaction: *F*_(2, 53)_ = 2.76, ns). Follow up comparisons indicate chronic stress-induced increases in iNOS mRNA in females but not males (p < .001, Fig 4D).

Stress altered Arg1 expression (*F*_(2, 55)_ = 7.22, p < .01), and this effect varied between males and females (effect of sex: *F*_(1, 55)_ = 0.38, ns; sex × stress interaction: *F*_(2, 55)_ = 6.80, p < .01). Unstressed females exhibited heightened Arg1 expression compared to unstressed males (p = .03). Acute stress induced Arg1 expression in males (p = .01), but decreased Arg1 expression in females (p = .01). Chronic stress reduced Arg1 mRNA in female but not male rats (p < .01, Fig 4E).

CX3CL1 expression was not affected by sex (*F*_(1, 54)_ = 0.27, ns) or stress (effect of stress: *F*_(2, 54)_ = 1.59, ns; sex × stress interaction: *F*_(2, 54)_ = 0.03, ns; Fig 4F). However, transcript levels of its receptor, CX3CR1, differed by stress (*F*_(2, 55)_ = 7.17, p < .01; effect of sex: *F*_(1, 55)_ = 1.29, ns; sex × stress interaction: *F*_(2, 55)_ = 2.85, ns). Acute stress increased CX3CR1 mRNA in males only (p < .01, Fig 4G).

There were no main effects of sex (*F*_(1, 55)_ = 0.21, ns) or stress (*F*_(2, 55)_ = 2.92, ns; sex × stress interactions, *F*_(2, 55)_ = 0.44, ns) on CD200 transcript (Fig 4H). Likewise, there were no main effects of sex (*F*_(1, 54)_ = 0.10, ns) or stress (*F*_(2, 54)_ = 0.81, ns) on CD200R expression. However, the effect of stress on CD200R transcript varied between males and females (*F*_(2, 54)_ = 3.17, p = .05). Follow up comparisons indicate greater CD200R expression in unstressed females compared to unstressed males (p = .03). Acute (p = .02) and chronic (p = .04) stress induced CD200R expression in males only (Fig 4I).

### 3.4 Microglial cell morphology and immune factor expression in dorsal hippocampus

**Fig 5:**
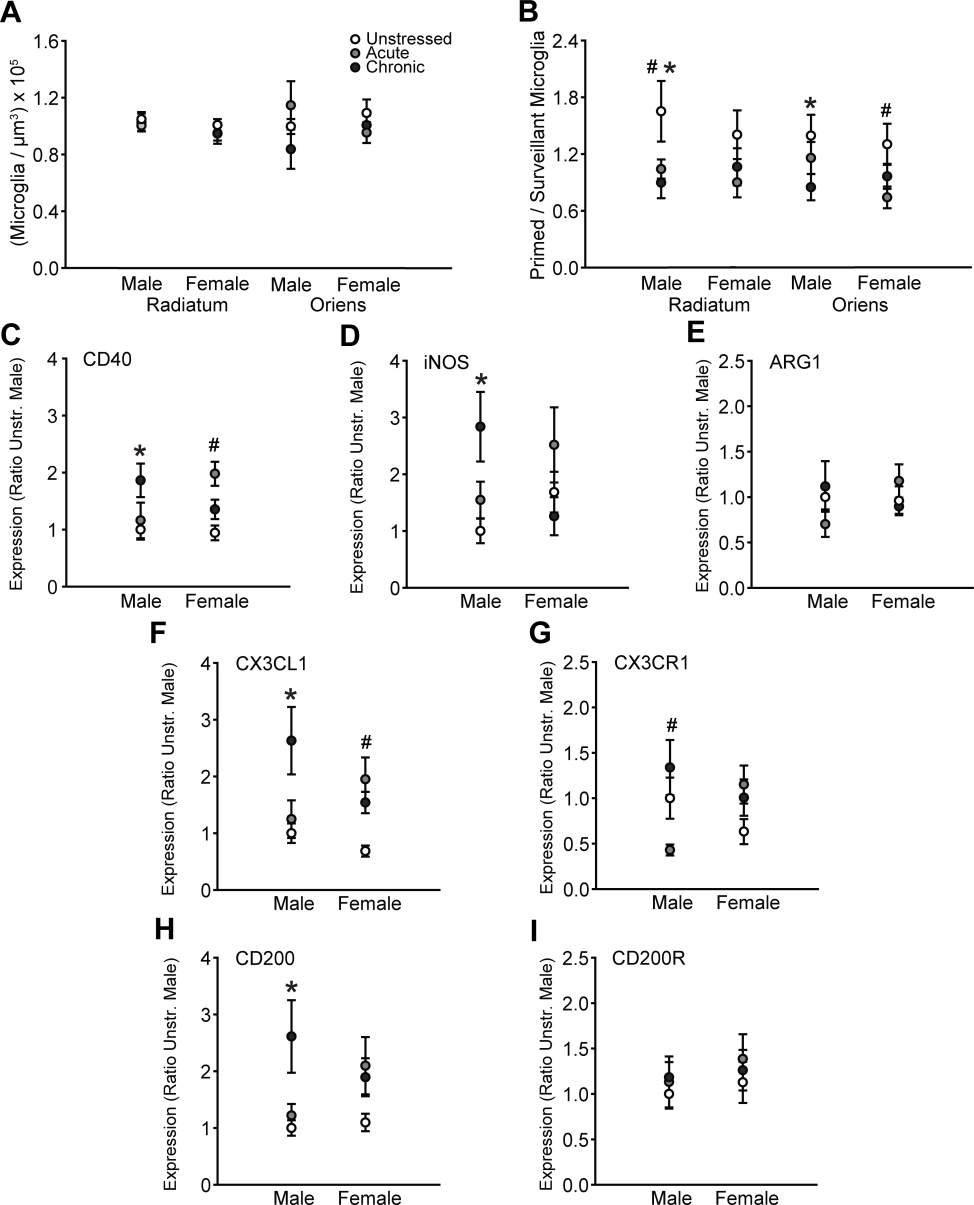
Sex and stress effects on microglial morphology and immune factor expression in dorsal hippocampus. A. Total microglial density based on estimated volume. There were no effects of sex or stress on microglial density in stratum radiatum or oriens of CA3. B. Acute and chronic stress reduced the proportion of primed to surveillant microglia in stratum radiatum of CA3 in males only. Acute stress reduced the proportion of primed to surveillant microglia in stratum oriens of CA3 in females, whereas chronic stress decreased the proportion of primed to surveillant microglia in males. C. Acute stress induced CD40 expression in females only. Chronic stress increased CD40 transcript in males. D. Chronic stress heightened iNOS expression in males only. E. There were no effects of sex or stress on Arg1 transcript. F. Acute stress increased CX3CL1 mRNA in females, whereas chronic stress induced CX3CL1 in males. G. Acute stress induced CX3CR1 expression in males. H. Chronic stress increased CD200 transcript in males. I. There were no effects of sex or stress on CD200R expression. ^#^p < .05, acute stress group compared to same-sex unstressed group. *p < .05, chronic stress group compared to same-sex unstressed group. Error bars indicate SEM. For graphs C-I, data are expressed relative to unstressed males.

Microglia were counted and morphologically classified in stratum radiatum and stratum oriens of the CA3 subregion of DHC. There were no effects of sex or stress on the total density of microglia in radiatum (sex: *F*_(1, 63)_ = 2.31, ns; stress: *F*_(2, 63)_ = 0.40, ns; sex × stress interaction: *F*_(2, 63)_ = 0.27, ns) or oriens (sex: *F*_(1, 62)_ = 1.10, ns; stress: *F*_(2, 62)_ = 0.13, ns; sex × stress interaction: *F*_(2, 62)_ = 1.51, ns; Fig 5A). However, morphological analyses revealed that stress influenced the proportion of primed to surveillant microglia in both regions (radiatum: main effect of stress, *F*_(2, 63)_ = 4.31, p = .02; main effect of sex, *F*_(1, 63)_ = 0.17; sex × stress interaction, *F*_(2, 63)_ = 0.49, ns; oriens: main effect of stress, *F*_(2, 64)_ = 3.83, p = .03; main effect of sex, *F*_(1, 64)_ = 0.83, ns; sex × stress interaction, *F*_(2, 64)_ = 1.17, ns; Fig 5B). Chronic stress reduced the proportion of primed to surveillant microglia in stratum radiatum (p = .01) and oriens (p = .02) in males only, whereas acute stress reduced the proportion of primed to surveillant microglia in stratum radiatum in males (p = .04) and stratum oriens in females (p = .04).

There was a main effect of stress (*F*_(2, 54)_ = 5.03, p = .01) on CD40 expression that differed between males and females (effect of sex: *F*_(1, 54)_ = 0.20, ns; sex × stress interaction: *F*_(2, 54)_ = 4.30, p = .02). Acute stress increased CD40 transcript in females (p < .01), whereas chronic stress increased CD40 expression in males (p < .01, Fig 5C)

There were no main effects of sex (*F*_(1, 53)_ = 0.01, ns) or stress (*F*_(2, 53)_ = 1.75, ns) on iNOS transcript. However, stress effects on iNOS expression varied between males and females (*F*_(2, 53)_ = 5.15, p < .01). Chronic stress increased iNOS transcript in males but not females (p < .01, Fig 5D). Arg1 expression was not affected by sex (*F*_(1, 54)_ = 0.26, ns) or stress (effect of stress: *F*_(2, 54)_ = 0.08, ns; sex × stress interaction: *F*_(2, 54)_ = 2.13, ns; Fig 5E).

Stress differentially affected CX3CL1 expression in males and females (main effect of stress: *F*_(2, 54)_ = 7.16, p = .002; main effect of sex: *F*_(1, 54)_ = 0.73, ns; stress × sex interaction: *F*_(2, 54)_ = 3.52, p = .04). Acute stress increased CX3CL1 transcript in females only (p = .01), whereas chronic stress induced CX3CL1 expression in males (p < .01, Fig 5F). Likewise, stress differentially affected CX3CR1 transcript in males and females (main effect of sex: *F*_(1, 54)_ = 0.00, ns; main effect of stress: *F*_(2, 54)_ = 2.08, ns; stress × sex interaction: *F*_(2, 54)_ = 4.26, p = .02). Acute stress reduced CX3CR1 mRNA in males only (p = .05, Fig 5G).

Levels of CD200 transcript were altered by stress (main effect of stress: *F*_(2, 54)_ = 5.45, p < .01; main effect of sex: *F*_(1, 54)_ = 0.08, ns; sex × stress interaction: *F*_(2, 54)_ = 2.29, ns). Chronic stress increased CD200 transcript in male but not female rats (p < .01, Fig 5H). However, CD200R expression did not differ across groups (Fig 5I; effect of sex: *F*_(1, 54)_ = 0.71, ns; effect of stress: *F*_(2, 54)_ = 0.44, ns; sex × stress interaction: *F*_(2, 54)_ = 0.08, ns).

### 3.5 Microglial morphological activation state across corticolimbic circuitry

**Fig 6:**
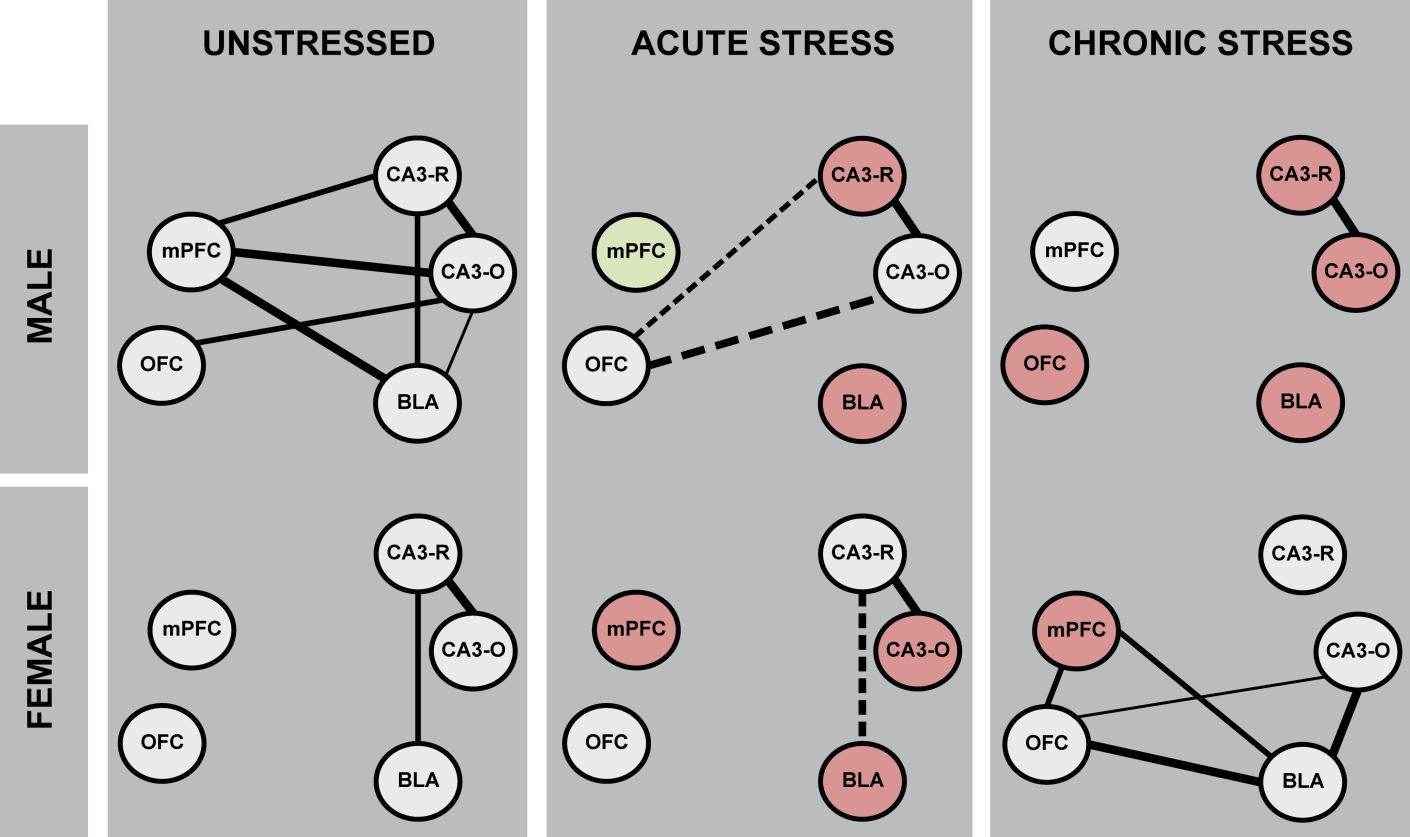
Sex differences in- and stress effects on- microglial morphological activation across corticolimbic brain regions. Associations between microglial morphological activation states across OFC, mPFC, BLA, and DHC were examined using Pearson correlation coefficients. Increased strength and/or number of associations would suggest greater microglial coupling, or decreased heterogeneity in microglial morphological activation state across corticolimbic brain regions. Microglial activation state is strongly correlated across corticolimbic brain regions in unstressed male- but not unstressed female- rats. Acute stress altered patterns of microglial morphological associations in both male and female rats. Remarkably, chronic stress reduced associations in microglial morphological activation state across corticolimbic circuitry in males, but induced associations in females. Green brain region nodes indicate an increase in microglial morphological activation state- and red nodes indicate a decrease in microglial morphological activation state as compared to same-sex unstressed animals. The strength of each association is represented in line weight (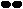, r = -1.00 –-.71; 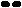, r = -0.70 –-0.61; 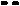, r = -0.60 –-0.50; 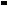, *r* = 0.50–0.60; 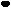, *r* = 0.61–0.70; 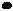, r = 0.71–1.00;); positive correlations are indicated with a solid line; negative correlations are indicated with a dashed line.

Microglial morphological activation state (i.e. the ratio of primed to surveillant microglia) was examined across OFC, mPFC, BLA, and the radiatum and oriens of the CA3 subregion of DHC using correlational analyses. Stronger correlations between structures would suggest less heterogeneity in microglial morphological activation state across these regions (i.e. greater coupling or synchrony in microglial morphology at a circuit level), while weaker correlations between structures would suggest greater heterogeneity. There were 9 potential relationships across corticolimbic circuitry, and 1 potential intrahippocampal relationship (i.e. CA3 radiatum-CA3 oriens). Seven strong and significant positive correlations were observed in unstressed male rats (OFC-CA3 oriens: *r*_(13)_ = 0.67, p = .01; mPFC-BLA: *r*_(12)_ = 0.82, p < .01; mPFC-CA3 radiatum: *r*_(12)_ = 0.67, p = .02; mPFC-CA3 oriens: *r*_(12)_ = 0.74, p < .01; BLA-CA3 radiatum: *r*_(13)_ = 0.67, p = .01; BLA-CA3 oriens: *r*_(13)_ = 0.60, p = .03; CA3 radiatum-CA3 oriens: *r*_(13)_ = 0.76, p < .01), whereas only two correlations were present in unstressed females (BLA-CA3 radiatum: *r*_(10)_ = 0.63, p = .05; CA3 radiatum-CA3 oriens: *r*_(10)_ = 0.87, p < .01; Fig 6). Thus, microglial morphological activation state is highly positively correlated across these corticolimbic structures in unstressed male but not female rats.

In males, acute stress induced a negative association between OFC and CA3-radiatum (*r*_(13)_ = -0.54, p = .05) and reversed the positive OFC-CA3 oriens relationship observed in unstressed rats (*r*_(13)_ = -0.63, p = .02); CA3 radiatum-CA3 oriens was correlated (*r*_(13)_ = 0.80, p < .01). In male rats exposed to chronic stress, microglial morphological activation state was correlated only within hippocampus (CA3 radiatum-CA3 oriens: *r*_(13)_ = 0.94, p < .001), but not across corticolimbic circuitry. These data indicate that both acute and chronic stress reduce the number of associations in microglial morphological state across corticolimbic circuitry in males.

In females, acute stress reversed the positive relationship between OFC-BLA as observed in unstressed rats (*r*_(10)_ = -0.63, p = .05); the relationship between CA3 radiatum-CA3 oriens was unaffected (*r*_(10)_ = 0.82, p < .01). In female rats exposed to chronic stress, 4 significant positive correlations were observed (OFC-mPFC: *r*_(11)_ = 0.67, p = .03; OFC-BLA: *r*_(10)_ = 0.83, p < .01; mPFC-BLA: *r*_(10)_ = 0.69, p = .03; BLA-CA3 oriens: *r*_(9)_ = 0.71, p = .03; OFC-CA3 oriens: *r*_(10)_ = 0.58, p = .08, ns). Microglial morphological activation state appears to be uncoupled between CA3 radiatum and CA3 oriens in chronic stress females (S2 Table). Thus, acute and chronic stress differentially affected microglial morphological activation state across corticolimbic circuitry in male and female rats. Chronic stress dramatically reduced morphological coupling in males, yet induced morphological coupling in females.

## 4. Discussion

These data demonstrate sex differences and differential effects of acute and chronic restraint stress on microglial density, morphology, and immune factor expression in OFC, BLA, and DHC. Expression of genes associated with cellular stress, microglial priming, and neuron-microglia communication varied between unstressed male and female rats. Moreover, associations between microglial morphological activation across OFC, mPFC, BLA, and DHC differed in males and females. Both acute and chronic stress differentially affected microglial activation state in males and females in all brain regions investigated (Table 2). Importantly, we also show that these sex-dependent stress effects differ within and across corticolimbic brain regions. Acute and chronic stress reduce the number of associations in microglial morphological activation state across corticolimbic circuitry in males (i.e. greater heterogeneity), whereas chronic- but not acute- stress induces microglial morphological coupling in females (i.e. less heterogeneity). These findings suggest the potential for microglia-mediated sex differences in stress effects on brain structure, function, and behavior across several stress-sensitive brain regions.

**Table 2:** Sex-dependent stress effects on microglia in corticolimbic circuitry.

	Orbitofrontal Cortex				Basolateral Amygdala				Dorsal Hippocampus			
Stress:	Acute		Chronic		Acute		Chronic		Acute		Chronic	
Sex:	♂	♀	♂	♀	♂	♀	♂	♀	♂	♀	♂	♀
Microglial density	**-**	**-**	**-**	**↓**	**-**	**-**	**-**	**-**	**-**	**-**	**-**	**-**
Microglial morphology	**-**	**-**	**↓**	**-**	**↓**	**↓**	**↓**	**-**	**↓**	**-**	**↓**	**-**
CD40 expression	**-**	**-**	**-**	**-**	**↑**	**-**	**-**	**↓**	**-**	**↑**	**↑**	**-**
iNOS expression	**-**	**↑**	**↑**	**↑**	**-**	**-**	**-**	**↑**	**-**	**-**	**↑**	**-**
Arg1 expression	**-**	**-**	**-**	**↑**	**↑**	**↓**	**-**	**↓**	**-**	**-**	**-**	**-**
CX3CL1 expression	**-**	**-**	**↑**	**-**	**-**	**-**	**-**	**-**	**-**	**↑**	**↑**	**-**
CX3CR1 expression	**↑**	**-**	**-**	**↑**	**↑**	**-**	**-**	**-**	**↓**	**-**	**-**	**-**
CD200 expression	**-**	**↑**	**-**	**↑**	**-**	**-**	**-**	**-**	**-**	**-**	**↑**	**-**
CD200R expression	**-**	**-**	**↑**	**-**	**↑**	**-**	**↑**	**-**	**-**	**-**	**-**	**-**

*Note*: **↑** (green) indicates increased microglial density, morphological activation, or gene expression compared to same-sex unstressed group; **↓** (red) indicates decreased microglial density, morphological activation, or gene expression compared to same-sex unstressed group.

### 4.1 Immune factor expression differs between unstressed male and female rats

Although no basal sex differences were detected in microglial density or morphology within OFC, BLA, or DHC, heterogeneity in microglial morphological activation state differed across corticolimbic circuitry in males and females. Males showed strongly positive correlations across mPFC, OFC, DHC, and BLA, while females showed strong relationships between microglial activation state in DHC and BLA only. This suggests that the factors controlling microglial activation are different in unstressed males and females. Further, given the role of microglia in regulating neuronal structure, plasticity, and function, the increased heterogeneity of activation across these regions in females could have profound implications for sex differences in the function of this circuit in regulating emotional and cognitive behavior.

Moreover, a number of genes were differentially expressed in unstressed male and female rats, and this varied across brain regions. In OFC, unstressed females exhibited significantly lower levels of CX3CL1 and CX3CR1 transcript compared to unstressed males. This could indicate a greater homeostatic demand for neuron-microglia inhibitory factor expression in OFC in male rats and, in turn, greater susceptibility to deficits induced by dysregulated neuron-microglia signaling. Moreover, lower inhibitory CX3CL1-CX3CR1 expression might indicate the presence of other, inhibitory pathways acting within OFC in females. For instance, microglia express estrogen receptors [50], and numerous reports indicate estradiol-mediated reductions in microglial activation in various disease models [50, 51].

Heightened CD40, Arg1, and CD200R expression was detected in BLA in unstressed female compared to male rats. Greater CD40 mRNA could indicate microglial priming or heightened neuroimmune activation [52], whereas greater CD200R and Arg1 expression might allow for neuron-microglia inhibitory signaling and reduced oxidative stress (i.e. a mixed, anti-inflammatory profile) [41, 53]. In contrast, no basal sex differences were identified in DHC. Together, these data indicate that resting immune profiles vary across sex and brain region. These patterns may contribute to region-specific differences in neural architecture and function, differences across corticolimbic circuitry more broadly, and sex differences in stressor and disease susceptibility.

### 4.2 Stress alters microglial cell morphology and immune factor expression in a brain region- and sex-specific manner

Chronic stress induces microglial activation, morphological remodeling, and process interaction with neuronal elements in various brain regions in male rodents [39, 54, 55]. For instance, stress increases the number of phagocytic cups and synaptic inclusions within microglial processes in mPFC. This is regulated by at least one neuron-microglia signal: colony stimulating factor 1 (CSF1) [54]. Alongside synaptic structure, evidence suggests that microglia are capable of remodeling neuronal architecture (e.g. dendritic length and branch number) [23]. Although correlative, our data are largely consistent with this notion.

Increased microglial activation is associated with reductions in dendritic length and spine density [23, 54], whereas microglial cell inhibition may allow for dendritic maintenance or growth [23]. In male rats, chronic stress produces either no change in microglial morphology or morphological activation in mPFC [24, 28, 30, 56], alters neuron-microglia signaling [54], and induces retraction of apical dendrites in pyramidal neurons [31]. The opposite pattern of stress-induced neuronal remodeling occurs in OFC (i.e. dendritic growth) [32]. In concordance with this, we found chronic stress-induced increases in inhibitory neuron-microglia CX3CL1 and CD200R expression, and reduced microglial morphological activation in OFC. Similarly, chronic stress increases apical dendritic length in pyramidal neurons in BLA in male rats (though this may depend on intensity or modality of stress) [57, 58], induces anxiety-like behavior [59] and, as reported herein, increases CD200R expression and decreases microglial morphological activation.

Further, chronic stress decreases apical dendritic arbor complexity in hippocampal CA3 pyramidal neurons in males. This effect is dependent on stressor duration, as 21 –but not 7 or 14 –days of stress induces dendritic remodeling [60]. Stress-induced microglial activation mirrors this time course: 10 days of stress reduces microglial morphological activation–perhaps a protective effect driven by the increased neuronal CX3CL1 and CD200 expression we have shown here–whereas 14 days of stress increases microglial activation [24]. With 21 days of stress, microglial phagocytic synaptic inclusions are present [39]. As dendritic remodeling in CA3 occurs between 14–21 days of stress, increased microglial activation at 14 days suggests that stress-linked changes in microglia precede changes in dendritic architecture.

Although little is known concerning sex differences in stress effects on neuronal morphology in OFC or BLA, our data are consistent with patterns of stress-induced dendritic remodeling in mPFC and DHC. Unlike male rats, chronic stress dramatically reduces microglial activation [30] and induces apical dendritic growth in pyramidal neurons in mPFC in females [11]. In DHC, chronic stress does not remodel apical dendrites [13] or alter microglial activation state in females.

While researchers have yet to examine sex-dependent stress effects on neuronal morphology in BLA, female rats may be resistant to stress-induced alterations in anxiety-like behavior [61]. Our findings align with these reports, and further predict no stress-linked changes in dendritic morphology in BLA in female rats. Stress decreased CD40 and Arg1 expression, and increased iNOS transcript. These alterations in oxidative stress pathways may indicate a mixed microglial phenotype [62]. Moreover, given sex-dependent shifts in stress-induced neurotransmission [63], such changes may serve a protective function in female rats by modulating the bioavailability of _L_-arginine and, in turn, glutamate and γ-aminobutyric acid (GABA) synthesis [64]. Note, however, that non-microglial cells (e.g. astrocytes, endothelial cells) can express iNOS and Arg1 [65]; thus, differences in these transcripts could reflect stress effects on non-microglial cell populations. Nonetheless, our data, together with recent findings [23, 39, 54], indicate that microglia-neuron interactions may contribute to sex differences in stress effects on brain architecture and behavior.

In addition to brain region specific effects, we identified different patterns of correlations of stress-induced microglial morphological activation across corticolimbic circuitry in males and females. In males, chronic stress reduced the number of associations between microglial activation across OFC, mPFC, BLA, and DHC, yet dramatically enhanced microglial morphological coupling within this circuit in females. These findings suggest that microglial morphological activation can shift at a circuit-relevant level, and may reflect a microglial response to- or regulation of- stress-linked corticolimbic signaling that differs between males and females. Additional studies investigating stress effects on microglial activation not just within brain regions, but also across circuitry may yield insight into circuit-level contributions to sex-specific stress effects on neural function and behavior.

### 4.3 Considerations and future directions

Female rats seem to exhibit increased variability in immune factor expression across multiple brain regions in this study compared to males. This may indicate activational effects of ovarian hormones in females, and further implicate ovarian hormones in the regulation of neuroimmune state [66, 67]. Although we were unable to analyze our data by estrous phase, future studies will address the role of gonadal hormones in microglial regulation, and their potential contributions to stress effects on- and sex differences in- microglial morphology and immune factor expression.

In this study, measures of gene expression aimed at neuron-microglia signaling pathways were derived from whole brain punches. As microglial density was largely unaffected by stress in males and females, alterations in microglia and macrophage-specific transcripts (i.e. CX3CR1 and CD200R) are likely the result of activational changes in neuroimmune cells present within each brain region assayed, and not microglial loss or migration. As the ligands CX3CL1 and CD200 are nearly exclusive to neuronal populations, stress-linked changes in their expression levels are unlikely to be non-neuronal in origin. In contrast, stress-induced changes in non-microglia specific transcripts (i.e. CD40, iNOS, and Arg1) may be the result of astroglial, endothelial, or pericytic activation–exclusively or in addition to microglial actions. Therefore, sex differences in- and stress effects on- these factors should be conceptualized as broad neuroimmune state in this study, and not microglial activation specifically. Future studies examining CD40, iNOS, and Arg1 in isolated microglia are warranted.

This study focused primarily on two neuron-microglia signaling pathways. This revealed circuit level sex differences in neuron-microglia factors, and the potential for sex dependent stress effects on neuron-microglia regulation. However, myriad immune molecules may be important in neuron-microglia interaction and microglial activation that could modulate sex differences in stress effects. For instance, interleukin (IL)-1β is associated with stress-induced macrophage recruitment and heightened anxiety-like behavior in males [68, 69], and female rats exhibit greater basal IL-1β and IL1r1 expression in cortical and hippocampal tissue compared to males [46]. Together, these studies suggest the potential for sex differences in stress-linked IL-1 signaling and, in turn, a pathway toward differential stress effects on neuronal function and behavior in males and females. Moreover, various immune genes are located on the X chromosome and could thus be differentially expressed across microglia in females versus males, including NFκB activating protein and chemokine receptor (CXCR) 3, both implicated in microglial activation and motility [34]. Such findings, concomitant with our data, underscore the need for additional studies examining stress effects on microglial phenotype, function, and immune signal expression in males and females.

## 5. Conclusion

We have demonstrated sex differences in- and sex-dependent stress effects on corticolimbic microglial morphology and immune factor expression. These effects comprise a complex assortment of changes in the expression neuroimmune factors and neuron-microglia inhibitory pathways. Notably, stress altered microglial morphology and immune factor expression in a duration- and brain region-specific manner, indicating that stress effects on corticolimbic microglia are not uniform across stress sensitive brain regions in either male or female rats. These findings agree with previous studies addressing brain region-dependent stress effects on neuronal architecture and behavior, and suggest important region-specific distinctions in pathways underlying microglial activation within- and across-sex. Moreover, stress altered heterogeneity of microglial morphological activation state across corticolimbic brain regions in a duration- and sex-specific manner. Such differences in the magnitude and direction of stress effects likely results in dysfunction of corticolimbic circuitry. Moreover, the brain regions examined in this study are critical in cognitive function and emotion regulation, and have been implicated in numerous stress-linked psychological disorders. Given the role of microglia in synaptic and dendritic maintenance, sex differences in stress effects on microglia may contribute to brain region-specific and sex-dependent alterations in neuronal structure, function, and behavior. Indeed, a recent study demonstrated chronic stress induced microglial remodeling of dendritic spines in mPFC in males but not females; this effect was regulated by neuron-microglia signaling [54]. As women are more vulnerable to inflammation-induced mood disturbances and various stress-linked psychological disorders [67, 70], differential stress effects on microglia and the neuroimmune milieu in males and females may underlie sex differences in susceptibility to stress-linked psychopathology.

## Supporting information

S1 File Body and adrenal weight data, and microglial morphology and immune factor expression data across orbitofrontal cortex, basolateral amygdala, and dorsal hippocampus. (ZIP)

S1 Table Pearson correlation coefficients: adrenal-weight-to-body-weight ratios, microglial density, and microglial morphology. (PDF)

S2 Table Pearson correlation coefficients: microglial morphology across corticolimbic circuitry. (PDF)
