# Supplementary material for: Behavioral stress alters corticolimbic microglia in a sex- and brain region-specific manner
Source: PLoS One. 2017 Dec 1;12(12):e0187631. doi: 10.1371/journal.pone.0187631 (PMC5711022; doi:10.1371/journal.pone.0187631)
Supplement: S1 Table — (PDF) [file pone.0187631.s002.pdf]

Supplementary Table 1. Pearson correlation coefficients: adrenal-weight-to-body-weight ratios, microglial density, and microglial morphology.

		Microglial Density					Microglial Morphology				
		OFC	mPFC	BLA	CA3-R	CA3-O	OFC	mPFC	BLA	CA3-R	CA3-O
Adrenal Weight	Male	-0.31	-0.01	0.05	-0.01	0.08	-0.26	0.14	-0.14	-0.34	-0.34
	Female	**-0.45**	-0.12	0.04	-0.32	-0.15	0.22	-0.19	-0.08	-0.21	-0.01

Note: Associations between adrenal-weight-to-body-weight ratios, microglial density, and microglial morphology were examined in unstressed and chronic stress male (n = 25-27 / analysis) and female (n = 20-21 / analysis) rats using Pearson correlation coefficients. Values are presented above, with significant associations marked in bold ($p \leq 0.05$).
