# Supplementary material for: Behavioral stress alters corticolimbic microglia in a sex- and brain region-specific manner
Source: PLoS One. 2017 Dec 1;12(12):e0187631. doi: 10.1371/journal.pone.0187631 (PMC5711022; doi:10.1371/journal.pone.0187631)
Supplement: S2 Table — (PDF) [file pone.0187631.s003.pdf]

Supplementary Table 2. Pearson correlation coefficients: microglial morphology across corticolimbic circuitry.

		Microglial Morphology				
		OFC	mPFC	BLA	CA3-R	CA3-O
Unstressed Male	OFC	1				
	mPFC	0.3	1			
	BLA	**0.82**	**0.82**	1		
	CA3-R	0.45	**0.67**	**0.67**	1	
	CA3-O	**0.67**	**0.74**	**0.6**	**0.76**	1
Acute Stress Male	OFC	1				
	mPFC	0.33	1			
	BLA	0.1	-0.3	1		
	CA3-R	**-0.54**	0.24	-0.31	1	
	CA3-O	**-0.63**	0.11	-0.46	**0.8**	1
Chronic Stress Male	OFC	1				
	mPFC	0.08	1			
	BLA	-0.28	0.01	1		
	CA3-R	-0.28	-0.01	-0.08	1	
	CA3-O	-0.38	-0.05	-0.11	**0.94**	1
Unstressed Female	OFC	1				
	mPFC	0.31	1			
	BLA	-0.29	-0.08	1		
	CA3-R	-0.1	0.48	**0.63**	1	
	CA3-O	0.15	0.54	0.56	**0.87**	1
Acute Stress Female	OFC	1				
	mPFC	0.39	1			
	BLA	-0.39	-0.23	1		
	CA3-R	0.28	-0.02	**-0.63**	1	
	CA3-O	0.01	-0.06	-0.51	**0.82**	1
Chronic Stress Female	OFC	1				
	mPFC	**0.67**	1			
	BLA	**0.83**	**0.69**	1		
	CA3-R	0.2	0.2	-0.11	1	
	CA3-O	0.58	0.42	**0.71**	0.41	1

Note: Microglial morphology was examined across corticolimbic circuitry in male (n = 12-14 / group) and female (n = 10-11 / group) rats using Pearson correlation coefficients. Values are presented above, with significant associations marked in bold ($p \leq 0.05$).
